# The burden of ambient temperature on years of life lost in Guangzhou, China

**DOI:** 10.1038/srep12250

**Published:** 2015-08-06

**Authors:** Jun Yang, Chun-Quan Ou, Yuming Guo, Li Li, Cui Guo, Ping-Yan Chen, Hua-Liang Lin, Qi-Yong Liu

**Affiliations:** 1 State Key Laboratory for Infectious Disease Prevention and Control, Collaborative Innovation Center for Diagnosis and Treatment of Infectious Diseases, National Institute for Communicable Disease Control and Prevention, Chinese Center for Disease Control and Prevention, Beijing 102206, China; 2 State Key Laboratory of Organ Failure Research, Department of Biostatistics, Guangdong Provincial Key Laboratory of Tropical Disease Research, School of Public Health and Tropical Medicine, Southern Medical University, Guangzhou 510515, China; 3 Division of Epidemiology and Biostatistics, School of Public Health, The University of Queensland, Brisbane, Queensland 4006, Australia; 4 Guangdong Provincial Institute of Public Health, Guangdong Provincial Center for Disease Control and Prevention, Qunxian Road, 160, Guangzhou 511430, China; 5 Climate Change and Health Center, Shandong University, Jinan 250012, China

## Abstract

Limited evidence is available on the association between temperature and years of life lost (YLL). We applied distributed lag non-linear model to assess the nonlinear and delayed effects of temperature on YLL due to cause-/age-/education-specific mortality in Guangzhou, China. We found that hot effects appeared immediately, while cold effects were more delayed and lasted for 14 days. On average, 1 °C decrease from 25^th^ to 1^st^ percentile of temperature was associated with an increase of 31.15 (95%CI: 20.57, 41.74), 12.86 (8.05, 17.68) and 6.64 (3.68, 9.61) YLL along lag 0–14 days for non-accidental, cardiovascular and respiratory diseases, respectively. The corresponding estimate of cumulative hot effects (1 °C increase from 75^th^ to 99^th^ percentile of temperature) was 12.71 (−2.80, 28.23), 4.81 (−2.25, 11.88) and 2.81 (−1.54, 7.16). Effect estimates of cold and hot temperatures-related YLL were higher in people aged up to 75 years and persons with low education level than the elderly and those with high education level, respectively. The mortality risks associated with cold and hot temperatures were greater on the elderly and persons with low education level. This study highlights that YLL provides a complementary method for assessing the death burden of temperature.

Numerous epidemiologic studies have examined the effects of cold and hot weathers on mortality and morbidity, particularly from cardiovascular and respiratory diseases^1^,^2^,^3^,^4^,^5^,^6^,^7^,^8^,^9^,^10^,^11^.

Temperature-mortality relationship was usually U or V-shaped, with excess mortality below and above a comfortable temperature^7^,^10^,^11^,^12^,^13^,^14^. The mortality effects of high temperature were usually reported to be acute, while the cold effects could last for several days, or even up to weeks^7^,^11^,^12^. And the elderly and persons with low socioeconomic status were found to be particularly vulnerable to ambient temperature. This information was important for policy-making for combating hazardous effects of temperature and protecting sensitive subpopulations.

Previous studies have mainly used mortality or morbidity as the health outcome to examine the temperature-health association^1^,^2^,^3^,^4^,^5^,^6^,^7^,^8^,^9^,^10^,^11^,^12^,^13^,^14^,^15^,^16^,^17^. However, these two indicators only considered the number of deaths or incidence and failed to take into account the differences in ages of the health outcomes, for example, a person who died at the age of 30 should pose different disease burden from another who died at the age of 80. At the same time, studies have suggested that the relative risk and the attributable deaths due to cold and hot temperatures had an increasing gradient with age, with smaller effects on the younger as compared to the elderly^11^,^18^. In fact, the younger had a longer life expectancy, then the burden of ambient temperatures on them would have more public health importance.

Years of life lost (YLL) is a measure of disease burden that takes account of life expectancy at death and gives higher weights to deaths at younger age. It is viewed as a more precise indicator of disease burden and has been intensively used to identify and prioritize causes of premature deaths around the world^19^. To date, very limited studies have examined the relationship of temperature and YLL^20^,^21^,^22^,^23^. As a first effort, Huang *et al.*^23^ reported the impact of the ambient temperature on YLL due to non-accidental mortality in Brisbane, Australia. Recently, Baccini and his colleagues reported that 55% of YLL related to heat wave per year happened among individuals younger than 75 years^20^. The evidence may be useful for guiding policy decision and resource allocation. However, temperature-YLL relationship among different cause-specific deaths and socioeconomic status is less clear; data of temperature-associated YLL are less available from developing countries.

This study was conducted to investigate the impact of hot and cold temperatures on YLL due to cause-specific mortality in Guangzhou, China, during 2003–2007, China; and to stratify the analyses by gender, age, and education level.

## Results

Table 1 shows the descriptive statistics for daily weather conditions, air pollutants and YLLs in Guangzhou. Mean temperature was 23.0 °C (ranging from 6.3 °C to 34.2 °C) between 2003 and 2007. The mean daily YLL were 781.5, 249.9 and 100.3 for deaths with non-accidental, cardiovascular and respiratory diseases, respectively. The average daily YLL were higher for male, people less than 75 years old and those with low education level than female, the elderly and person with high education level, respectively.

**Figure 1:**
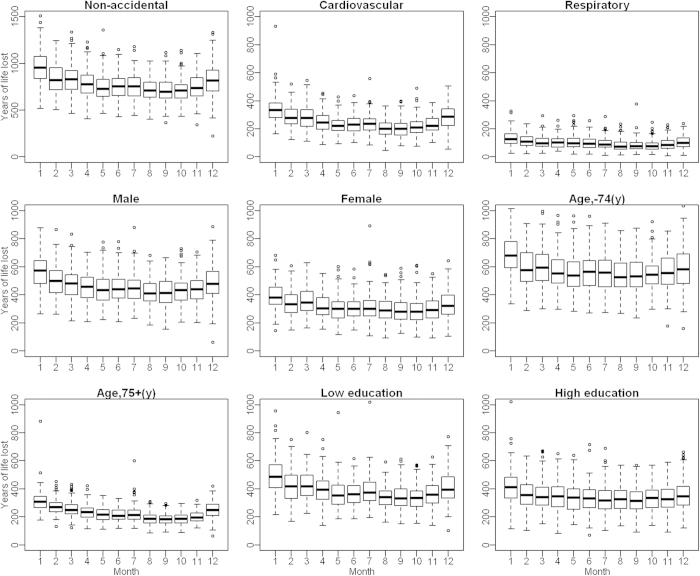
Boxplots of monthly YLL in Guangzhou, China during 2003–2007.

Figure 1 shows the boxplots of monthly YLL. YLL generally had a significant seasonal trend, with higher in the cold months (November to February) than the hot months (May to September). There was a small peak in YLL in June or July.

**Figure 2:**
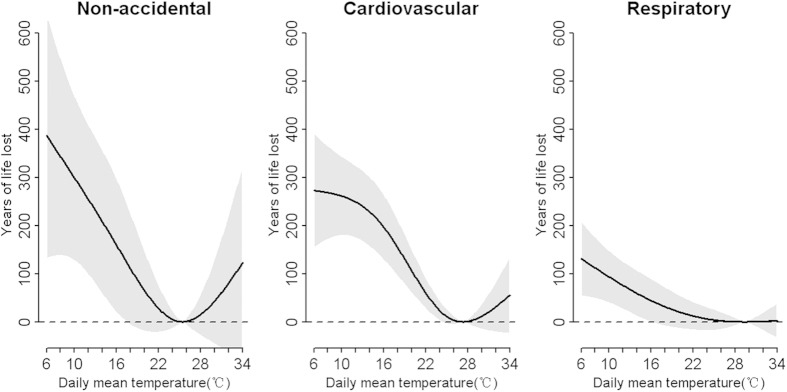
The dose-response curves of temperature and YLL due to cause-specific deaths across lag0–30, in Guangzhou, China during 2003–2007. The minimum-YLL temperature was 25.4 °C, 27.3 °C, and 29.4 °C for non-accidental, cardiovascular and respiratory disease, respectively.

Figure 2 shows the exposure-response curves between daily mean temperature and YLL. All curves are U- or reverse J-shaped. The temperatures of 25.4 °C, 27.3 °C, and 29.4 °C were related with minimum YLL for non-accidental, cardiovascular and respiratory diseases, respectively.

**Figure 3:**
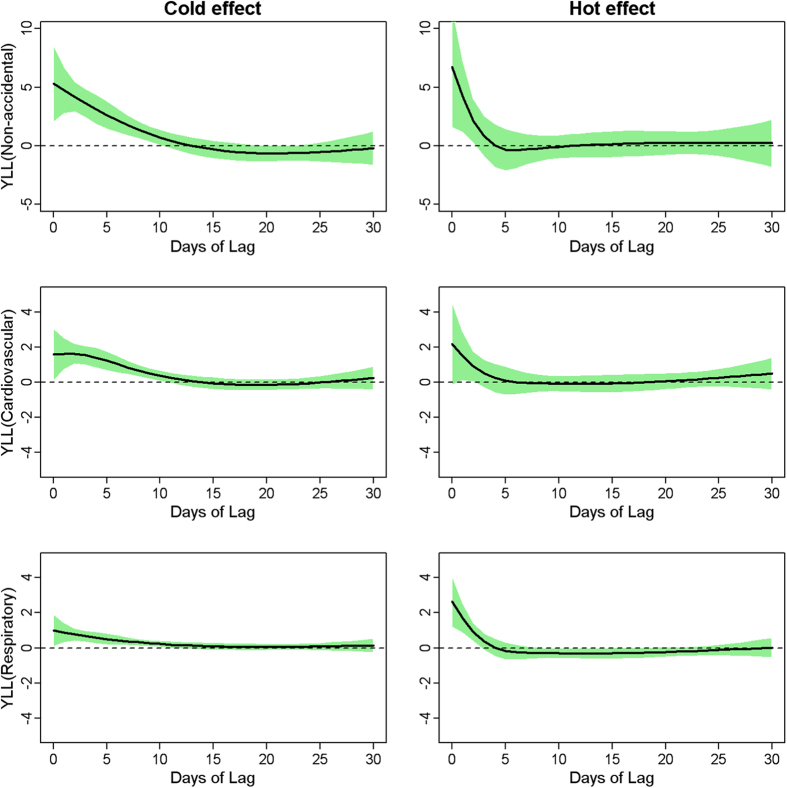
Cold and hot effect on YLL due to mortality categories along days of lag in Guangzhou, China during 2003–2007. Cold effect was presented by YLL changes per 1 °C decrease from 25^th^ percentile temperature to 1^st^ percentile of temperature; hot effect was presented by YLL changes per 1 °C increase from 75^th^ percentile temperature to 99^th^ percentile of temperature.

Figure 3 shows the lag structures of cold and hot effects on YLL. In general, the effects of hot temperature appeared acutely and lasted for 4 days, while cold effects peaked at 2 days after exposure and declined slightly with duration of 14 days. Therefore, we present the accumulative hot and cold effects along 14 days. Moreover, not statistically significant harvesting effect was observed for hot and cold temperature on YLL due to mortality categories, respectively.

Table 2 shows the cumulative effect of temperature on non-accidental, cardiovascular and respiratory deaths stratified by individual characteristics at lag 0–14. The cumulative cold effects along lag 0–14 days were associated with an increase of 31.15 (95%CI: 20.57, 41.74), 12.86 (8.05, 17.68) and 6.64 (3.68, 9.61) YLL for non-accidental, cardiovascular and respiratory diseases, respectively. The corresponding estimates of cumulative hot effects were 12.71 (−2.8, 28.23), 4.81 (−2.25, 11.88) and 2.81 (−1.54, 7.16), respectively. Generally, the cumulative cold effects were stronger than hot effects. Cold effects were significantly associated with YLL increase for all subgroups, while hot temperatures only statistically impact women, the elderly and those with low education level. Effect estimates of cold and hot -related YLL were higher in people aged lower than 75 years and persons with low education level than the elderly and those with high education level.

The estimates of relative risk temperature-associated of mortality revealed greater effects of hot temperature among females than among males, with opposite trend for cold effects. The elderly, those with low education level were at higher relative risk of mortality associated with both hot and cold temperatures than the youth and people with high education level (Table 3).

Sensitivity analyses were performed to check whether the results were robust to the specification of parameters in the model. Supplemental material Figure S1 revealed that the residuals of the model for non-accidental mortality were approximately normally distributed and independent over time. Supplemental Material Figure S2 showed that the effect estimates for non-accidental mortality were stable when using 6 or more degrees of freedom per year for time and 3-6 df for air pollutants, atmospheric pressure and relative humidity, and the maximum lag from 21 to 30 days for mean temperature.

## Discussion

To the best of our knowledge, this is the first study to examine the relationship between ambient temperature and YLL in China. We found that cold and hot temperatures have significant impacts on YLL due to non-accidental and cardio-respiratory diseases. The hot effects appeared to be acute and lasted only for 4 days, while cold effects were delayed but persisted for 14 days. Generally, the cumulative cold effects were stronger than those of hot temperatures. Cold and hot effects-related YLL were higher in people aged up to 75 years and persons with low education level than those older than 75 years and those with high education level.

The most of previous studies have inverstigated the relative risk of temperature-related mortality. However, mortality risk does not take deaths happening at different ages into account and the relative risk is believed to be heavily driven by short term mortality displacement^21^,^24^. In contrast, YLL is a useful measure for assessing premature death. Thus, it has been argued that YLL is more informative indicator to assess mortality impact related to temperature exposure for accounting for the life expectancy^22^,^23^. Understanding the impact of temperature on YLL will be helpful for health risks evaluation again other exposures.

Cold and hot weather were significant assocaited with mortality risk^2^,^7^,^11^ and YLL increase^21^,^22^. Exposure to cold temperatures is associated with changes in blood pressure, and an increase in blood viscosity, levels of red blood cell count and peripheral vasoconstriction^25^. Exposure to high temperature may place stress on the thermoregulatory system, and causes increases in blood viscosity, blood cholesterol levels and salt depletion. Compared with investigation of Baccini and colleagues^20^, no apparent harvesting effects of temperature on YLL were observed in Guangzhou, China. This finding was also in agreement with study of Huang *et al.*^22^. We speculated that the harvesting effect may be modified by different climates, regions and socioeconomic characteristics. For example, Baccini *et al.*^20^ reported that harvesting effect was more pronounced in the Northcontinental than in the Mediterranean cities.

It is important to understand the lag pattern of temperature effects on YLL, because the information can be used to develop early response plan for cold and hot temperatures by healthcare promoters and local communities. This study demonstrated that the effects of heat on YLLs were acute with a few days lasting. But the cold effects were somewhat delayed and persisted up to 14 days, which was consistent with the study of Huang and his colleagues^22^. Therefore, promptly and proper preventive actions are recommended for reducing harmful effects of high temperature in summer, while protecting measures against cold temperature in winter should be lasting for days after the cold days.

We found that cold temperatures significantly impact the YLLs due to almost all mortality categories in Guangzhou, China. And the effects of cold temperatures were stronger than those of hot temperatures. This information indicated that cold weather is till the main disease burden for populations in subtropical regions, comparing to the hot weather, even in context of global warming scenario. And it is a need to strengthen the awareness of combating cold exposure in the public even in subtropical region. A collaborative mechanism across multi-sectors and institutions is required to better monitor weather conditions in these regions, especially for the cold weather.

Many epidemiological studies have provided evidence that susceptibility to cold and hot temperatures is modified by age, gender and education level^9^,^11^,^18^. Consistent with previous temperature-mortality studies, our results confirmed that the elderly were at higher mortality risk of both hot and cold temperatures than young group. This may be due to relatively poorer physiological adaptation and pre-existing chronic diseases among the elderly. However, using YLL as health outcome presented a different impact pattern along age, with greater YLL associated with cold and hot temperatures among the youth, which is contrast to the findings of higher relative risk of mortality among the elderly. This difference may be helpful for decision-making. Taking no account of deaths happening at very different ages may distort resources allocation. Thus, evidence on YLL-temperature relationship will be useful for policy-makers on risk management. Moreover, with increasing societal resources on preventive cares for the elderly, the protecting measures for the youth to combat the harmful effects of ambient temperatures should not be ignored.

Consistent with temperature-mortality studies, we found that gender might be an effect modifier of the relationship between daily mean temperature and YLL. Effect estimate of YLL due to cold temperatures was higher among males than females, with opposite trend for hot temperatures. The difference may be due to the socioeconomic factors and biological differences between the two genders. Our results also indicated that both cold and hot effects-associated YLL were higher in persons with low education than those with higher education level, trend of which is similar to temperature impacts on mortality between education levels. Education level is one of the most important indicators of one’s overall socioeconomic status (SES). People with low SES may be associated with poorer health condition, limited access to health care, poor housing environment, lack of preventive knowledge and behavior patterns such as smoking, which may contribute to their susceptibility of ambient temperature.

Some limitations needed to be considered. Firstly, the data are only from one city, so it should be cautious to generalize the findings to other geographic areas and other climates. Secondly, as many studies, we used the data on temperature from fixed sites rather than measuring individual exposure. It may bring about measurement errors since indoor temperature is not closely correlated with outdoor temperature due to the use of air conditioning. Furthermore, we did not control for the impact of ozone, as the data were unavailable to this analysis. Previous studies reported that effects of hot temperature were slightly reduced after controlling for ozone, while the cold effects were not changed. Future research needs to look into this issue.

In summary, our study provides new insights on the health burden of ambient extreme temperatures in China. This study highlights that both cold and hot temperatures have significant impacts on YLL. The effects of hot temperatures appeared immediately, while cold effects were more delayed and last for 14 days. Cold and hot temperatures-associated YLL were higher in the youth and people with low education level than the elderly and those with high eudcation level, respectively. Special interventions and measures should target on these subpopulations to reduce temperature-related YLL.

## Methods

### Data collection

Guangzhou is the capital and largest city of Guangdong Province, China. Located on the Pearl River, it is the most populous city in South China, with a population of 12.79 million. It has a subtropical climate with hot summer but mild winter.

Our study includes six central urban districts of Guangzhou where there are 7.7 million permanent residents. Individual death data were obtained from the Guangzhou Bureau of Health during 1 January 2003 and 31 December 2007, with a total of 117,233 registered deaths. The data comprised birth date, date of death, cause of death, gender, and education level. Causes of death were coded according to the International Classification of Diseases, the tenth version (ICD-10). We examined non-accidental (A00-R99), cardiovascular (I00-I99) and respiratory (J00-J99).

Chinese national life tables from the years 2000 and 2009 were obtained from World Health Organization (WHO)^26^. Life expectancies for 2003–2007 were averaged from the years 2000 and 2009 (Supplemental Material, Table S1). We calculated YLL by matching age and sex to the life tables for each death. Daily YLL were calculated by summing the YLL for all deaths on the same day. We stratified the sums by death causes, gender, age group (0–74 and 75 + years) and education level (low education: primary school or lower; high education: secondary or higher). This method of YLL calculation has been applied in previous investigations^22^,^23^,^24^,^27^.

Daily meteorological data were provided by China Meteorological Data Sharing Service System. We used daily mean temperature, relative humidity and barometric pressure from Guangzhou Weather Station located near the city center at 23.10°N latitude and 113.20°E longitude, which is the only national basic weather station in Guangzhou (Supplemental Material, Figure S3). Daily air pollution data were provided by Guangzhou Bureau of Environmental Protection, including particulate matter less than 10 μm in aerodynamic diameter (PM_10_), nitrogen dioxide (NO_2_) and sulphur dioxide (SO_2_). In this study, we selected seven fixed-site air monitoring stations located in the six central urban districts under study. The daily average concentration of each pollutant for these stations was computed using the centering method^28^,^29^.

### Data analysis

Distributed lag non-linear model, proposed by Gasparrini *et al.*, is flexible enough to examine non-linear exposure-response relationship and delayed effect simultaneously, after accounting for the strong collinearity of exposure variable. The daily YLL follows a normal distribution (Supplemental Material, Figure S4). Thus, we applied a general linear regression model with distributed lag non-linear model to assess the non-linear and delayed effects of ambient temperature on YLL, as following:


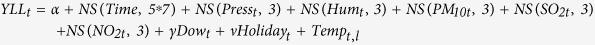


where *YLL*_*t*_ is the observed daily YLL at day *t* (*t* = 1,2,3…1826); *α* is the intercept; *NS*(.) means a natural cubic spline; 7 degrees of freedom (df) per year for time were used to control long-term trend and seasonality, and 3 df for air pollutants, barometric pressure and relative humidity was used to control for their potential confounding effects, respectively*; Dow*_*t*_ and *Holiday*_*t*_ are day of the week and public holidays, represented as categorical variables. *Temp*_*t,l*_ is a two-dimensional natural spline for temperature and the lag days, with a maximum lag of *l*.

To appraise prolonged effects of cold temperature and potential harvesting effects of hot temperature, we chose 30 days as the maximum lag for temperature, in line with the study of Baccini *et al.*^20^. The degrees of freedom (df) for temperature and lag were selected by the minimum value of the average of Akiake information criterion (AIC). The final df was 4 for temperature and 4 for lag with lowest AIC value of 17777.36.

We first flexibly plotted the overall association between YLL and temperature along lag 0–30, from which the temperature with lowest YLL was extracted as the minimum-YLL temperature. We then calculated the absolute number of YLL change comparing a percentile to another percentile of temperature, and provided the estimates of average change in YLL per 1 °C temperature change over this range. For cold effects, we calculated the average number of YLL change per 1 °C decrease from the 25^th^ percentile of temperature to 1^st^ percentile of temperature; for the hot effects, we calculated the average number of YLL change per 1 °C increase from the 75^th^ percentile of temperature to 99^th^ percentile of temperature. This method of assessing cold and hot effects had been applied in previous study^30^.

To compare the standard analysis of mortality and the analysis of YLL, we also presented the relative risk of mortality associated with 1 °C decrease from the 25^th^ percentile to 1^st^ percentile of temperature (cold effects) and 1 °C increase from 75^th^ percentile to 99^th^ percentile of temperature (hot effects).

Sensitivity analyses were carried out by changing df (6–10 per year) for time to control for season, df (3–6) for air pollutants and atmospheric pressure, and the maximum lag from 21 to 30 days for daily mean temperature.

To check the adequacy of all models, we used an autocorrelation function to examine if the residuals were independent and randomly distributed over time. All statistical analyses and modeling were completed in R version 3.1.0. The dlnm package was used to perform the distributed lag non-linear models. For all statistical tests, two-tailed P < 0.05 were considered statistically significant.

## Additional Information

**How to cite this article**: Yang, J. *et al.* The burden of ambient temperature on years of life lost in Guangzhou, China. *Sci. Rep.* **5**, 12250; doi: 10.1038/srep12250 (2015).

## Supplementary Material

Supplementary Information

## Figures and Tables

**Table 1:** Descriptive statistics of daily weather conditions and years of life lost from 2003 to 2007 in Guangzhou, China.

**Variables**	**Minimum**	**1%**	**25%**	**Median**	**75%**	**99%**	**Maximum**	**Mean**	**SD**
Daily meteorological measures									
Maximum temperature(°C)	7.2	11.0	23.4	28.8	32.6	37.2	39.1	27.6	6.2
Mean temperature(°C)	6.3	8.2	18.6	24.4	28.0	32.0	34.2	23.0	6.1
Minimum temperature(°C)	2.1	5.5	15.3	21.1	25.0	28.5	30.4	19.8	6.2
Relative humidity (%)	20.0	35.0	64.0	72.0	80.0	93.0	97.0	80.0	12.9
Atmospheric pressure(hPa)	988.7	994.4	1003.4	1008.5	1014.0	1022.6	1027.2	1008.7	6.8
Daily concentrations of air pollutants									
PM_10_(μg/m^3^)	7.0	19.1	52.1	80.0	114.6	253.6	370.1	88.2	48.5
NO_2_(μg/m^3^)	24.7	29.4	48.0	65.8	89.9	185.1	281.3	73.2	34.0
SO_2_(μg/m^3^)	6.1	8.3	29.3	49.7	80.3	180.8	237.3	59.3	39.6
Daily years of life lost									
Non-accidental mortality	224.6	464.5	665.6	765.3	877.1	1247.4	2699.0	781.5	170.4
Cardiovascular mortality	44.8	105.9	194.3	240.5	297.5	457.7	933.2	249.9	80.7
Respiratory mortality	8.0	24.1	67.2	92.9	126.2	239.2	378.9	100.3	45.9
Gender									
Male	62.3	235.6	380.2	454.6	531.8	772.4	1543.3	461.4	117.4
Female	90.8	143.3	251.2	309.0	376.9	574.1	1155.7	320.0	98.2
Age(years)									
0-74	159.7	297.3	476.8	565.3	663.8	945.7	1882.8	576.1	143.3
75+	64.9	117.2	183.5	219.6	264.2	402.8	883.5	227.8	63.8
Education level									
Low education	102.4	186.6	311.9	373.9	458.6	698.0	1524.9	388.9	113.4
High education	69.9	137.2	273.1	338.6	409.1	628.3	1021.5	346.3	103.9
Number of daily deaths									
Non-accidental mortality	20	39	52	59	69	99	233	61.5	13.6
Cardiovascular mortality	6	11	19	23	28	44	102	23.8	7.4
Respiratory mortality	2	4	8	11	14	24	46	11.5	4.5
Gender									
Male	8	19	29	33	39	57	127	34.2	8.0
Female	11	14	22	27	32	47	106	27.3	7.6
Age(years)									
0–74	9	16	25	28	33	46	103	29.0	6.6
75+	11	17	26	31	38	60	130	32.5	9.4
Education level									
Low education	14	20	30	36	43	65	159	37.5	10.2
High education	6	9	16	20	24	35	61	20.0	5.6

Note. low education: primary school or lower; high education: secondary or higher.

**Table 2:** The cumulative cold and hot effects on YLL due to non-accidental, cardiovascular and respiratory mortality across lag0-14 stratified by individual characteristics.

	**Non-accidental**		**Cardiovascular**		**Respiratory**	
	**Cold effect(95%CI)**	**Hot effect(95%CI)**	**Cold effect(95%CI)**	**Hot effect(95%CI)**	**Cold effect(95%CI)**	**Hot effect(95%CI)**
All	31.15 (20.57,41.74)*	12.71 (−2.80, 28.23)	12.86 (8.05, 17.68)*	4.81 (−2.25, 11.88)	6.64 (3.68, 9.61)*	2.81 (−1.54, 7.16)
Gender						
Male	17.81 (10.08,25.55)*	2.59 (−8.76, 13.93)	8.18 (4.58, 11.78)*	0.3 (−4.98, 5.57)	4.07 (1.81, 6.32)*	0.92 (−2.39, 4.23)
Female	13.34 (6.90,19.79)*	10.12 (0.68, 19.57)*	4.68 (1.64, 7.73)	4.52 (0.06, 8.98)*	2.57 (0.68, 4.46)*	1.88 (−0.89, 4.66)
Age (years)						
0−74	18.83 (9.15,28.51)*	6.96 (−7.22, 21.15)	7.76 (3.45, 12.08)*	1.05 (−5.27, 7.37)	2.19 (−0.47, 4.85)*	1.49 (−2.42, 5.38)
75+	13.46 (10.10,16.82)*	5.97 (1.04, 10.89)*	6.47 (4.31, 8.62)*	3.42 (0.25, 6.58)*	4.46 (3.06, 5.87)*	1.16 (−0.90, 3.22)
Education Level						
Low education	14.76 (7.71,21.80)*	15.76 (5.43, 26.10)*	7.14 (3.93, 10.35)*	4.63 (−0.07, 9.34)	4.63 (2.22, 7.03)*	2.11 (−1.42, 5.64)
High education	14.06 (7.16,20.96)*	−5.54 (−15.66, 4.58)	5.13 (1.83, 8.43)*	−0.77 (−5.61, 4.07)	1.54 (−0.12, 3.19)	−0.03 (−2.46, 2.4)

Note. *P<0.05; cold effect presentsYLL changes per 1 °C decrease from 25th percentile temperature to 1^st^ percentile of temperature; hot effect presents YLL changes per 1 °C increase from 75^th^ percentile temperature to 99^th^ percentile of temperature; low education: primary school or lower; high education: secondary or higher.

**Table 3:** The cumulative relative risk of cold and hot effects on mortality due to non-accidental, cardiovascular and respiratory mortality across lag0-14 stratified by individual characteristics.

	**Non-accidental**		**Cardiovascular**		**Respiratory**	
	**Cold effect(95%CI)**	**Hot effect(95%CI)**	**Cold effect(95%CI)**	**Hot effect(95%CI)**	**Cold effect(95%CI)**	**Hot effect(95%CI)**
All	1.04 (1.03,1.05)*	1.03 (1.01, 1.04)*	1.04 (1.03, 1.06)*	1.04 (1.02, 1.07)*	1.06 (1.04, 1.09)*	1.04 (1.00, 1.07)*
Gender						
Male	1.05 (1.03,1.06)*	1.01 (0.99, 1.03)	1.05 (1.03, 1.07)*	1.03 (0.99, 1.06)	1.07 (1.04, 1.10)*	1.02 (0.98, 1.07)
Female	1.04 (1.02,1.05)*	1.04 (1.02, 1.07)*	1.03 (1.01, 1.05)*	1.06 (1.02, 1.09)*	1.06 (1.03, 1.09)*	1.05 (1.00, 1.11)*
Age (years)						
0–74	1.04 (1.02,1.05)*	1.00 (0.98, 1.02)	1.05 (1.02, 1.07)*	1.01 (0.97, 1.04)	1.05 (1.01, 1.1)*	1.00 (0.94, 1.07)
75+	1.05 (1.03,1.06)*	1.05 (1.02, 1.07)*	1.04 (1.02, 1.06)*	1.06 (1.03, 1.1)*	1.07 (1.04, 1.09)*	1.05 (1.01, 1.09)*
Education Level						
Low education	1.04 (1.03,1.05)*	1.03 (1.01, 1.05)*	1.04 (1.02, 1.06)*	1.05 (1.02, 1.08)*	1.06 (1.03, 1.08)*	1.02 (0.98, 1.06)
High education	1.04 (1.02,1.06)*	1.00 (0.98, 1.03)	1.05 (1.02, 1.08)*	1.01 (0.97, 1.05)	1.06 (1.02, 1.11)*	1.06 (0.98, 1.14)

Note. *P<0.05; cold effect presents relative risk for per 1 °C decrease from 25th percentile temperature to 1^st^ percentile of temperature; hot effect presents relative risk for per 1 °C increase from 75^th^ percentile temperature to 99^th^ percentile of temperature; low education: primary school or lower; high education: secondary or higher.
