# Supplementary material for: The burden of ambient temperature on years of life lost in Guangzhou, China
Source: Sci Rep. 2015 Aug 6;5:12250. doi: 10.1038/srep12250 (PMC4527090; doi:10.1038/srep12250)
Supplement: Supplementary Information [file srep12250-s1.pdf]

Supplemental Materials

The burden of ambient temperature on years of life lost in Guangzhou, China

Jun Yang[1,5, *], Chun-Quan Ou[2, *], Yuming Guo[3], Li Li[2], Cui Guo[2], Ping-Yan Chen[2], Hua-Liang Lin[4], Qi-Yong Liu[1,5*]

[1] State Key Laboratory for Infectious Disease Prevention and Control, Collaborative Innovation Center for Diagnosis and Treatment of Infectious Diseases, National Institute for Communicable Disease Control and Prevention, Chinese Center for Disease Control and Prevention, Beijing 102206, China.

[2] State Key Laboratory of Organ Failure Research, Department of Biostatistics, Guangdong Provincial Key Laboratory of Tropical Disease Research, School of Public Health and Tropical Medicine, Southern Medical University, Guangzhou 510515, China.

[3] Division of Epidemiology and Biostatistics, School of Population Health, The University of Queensland, Brisbane, Queensland 4006, Australia.

[4] Guangdong Provincial Institute of Public Health, Guangdong Provincial Center for Disease Control and Prevention, Qunxian Road, 160, Guangzhou 511430, China.

[5] Climate Change and Health Center, Shandong University, Jinan 250012, China.

[*] These authors contributed equally to this work.

Correspondence and requests for materials should be addressed to Q.Y.L. (liuqiyong@icdc.cn)

OUTLINE

Title	Page
**Table S1:** Life expectancy for Chinese population.	**3**
**Figure S1:** Checking the residual of the model for non-accidental mortality.	**4**
**Figure S2:** Sensitivity analyses by changing degrees of freedom (df) (6-10 per year) for time to control for season, df (3-6) for relative humidty, air pollutants and atmospheric pressure, and maximum lag from 21-30 days for mean temperature.	**5**
**Figure S3:** Map of districts in Guangzhou, China.	**6**
**Figure S4:** Histogram of daily years of list lost due to mortality categories in Guangzhou, China during 2003-2007.	**7**

Table S1: Life expectancy for Chinese population.

Age	Life expectancy for Male (year)			Life expectancy for Female (year)		
	2000	2009	Mean	2000	2009	Mean
0	69.9	72.2	71.1	72.6	75.8	74.2
1	70.6	72.2	71.4	74.2	76.3	75.3
5	67.1	68.4	67.8	70.7	72.5	71.6
10	62.3	63.5	62.9	65.9	67.6	66.8
15	57.4	58.6	58.0	61.0	62.7	61.9
20	52.7	53.8	53.3	56.1	57.8	57.0
25	48.1	49.1	48.6	51.3	52.9	52.1
30	43.4	44.4	43.9	46.5	48.1	47.3
35	38.7	39.7	39.2	41.7	43.2	42.5
40	34.1	35.0	34.6	37.0	38.5	37.8
45	29.6	30.4	30.0	32.4	33.8	33.1
50	25.2	26.0	25.6	27.9	29.2	28.6
55	21.1	21.8	21.5	23.5	24.8	24.2
60	17.2	17.9	17.6	19.3	20.5	20.0
65	13.8	14.3	14.1	15.4	16.4	16.0
70	10.7	11.2	11.0	11.9	12.7	12.3
75	8.1	8.4	8.3	8.8	9.5	9.2
80	5.9	6.2	6.1	6.4	6.9	6.7
85	4.3	4.5	4.4	4.6	4.8	4.7
90	3.1	3.2	3.2	3.2	3.4	3.3
95	2.3	2.3	2.3	2.3	2.4	2.4
100	1.7	1.8	1.8	1.8	1.8	1.8

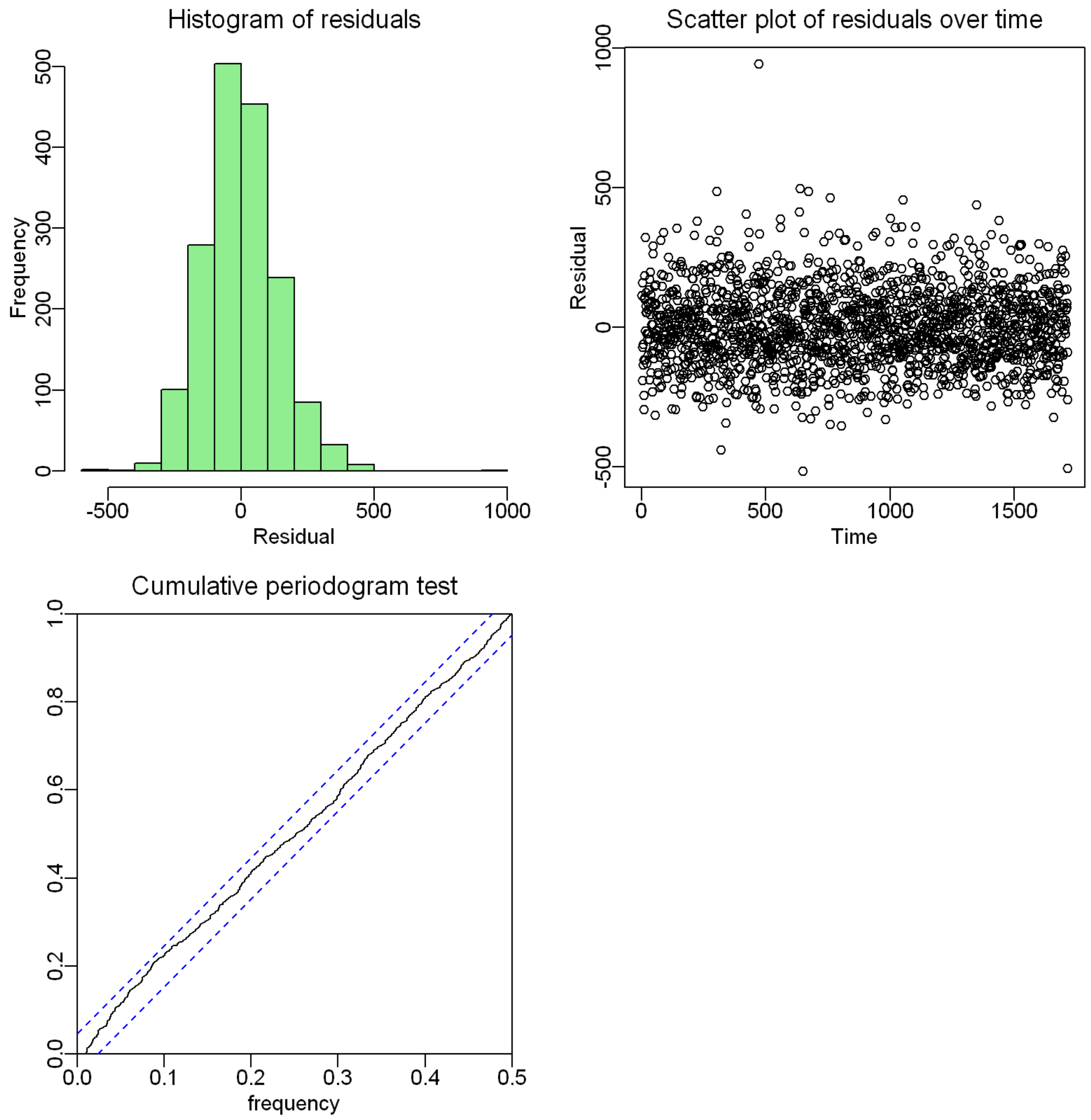

Figure S1: Checking the residuals of the model for non-accidental mortality. The residuals were nornally distributed. The cumulative periodogram test shows the independence of the residuals over time.

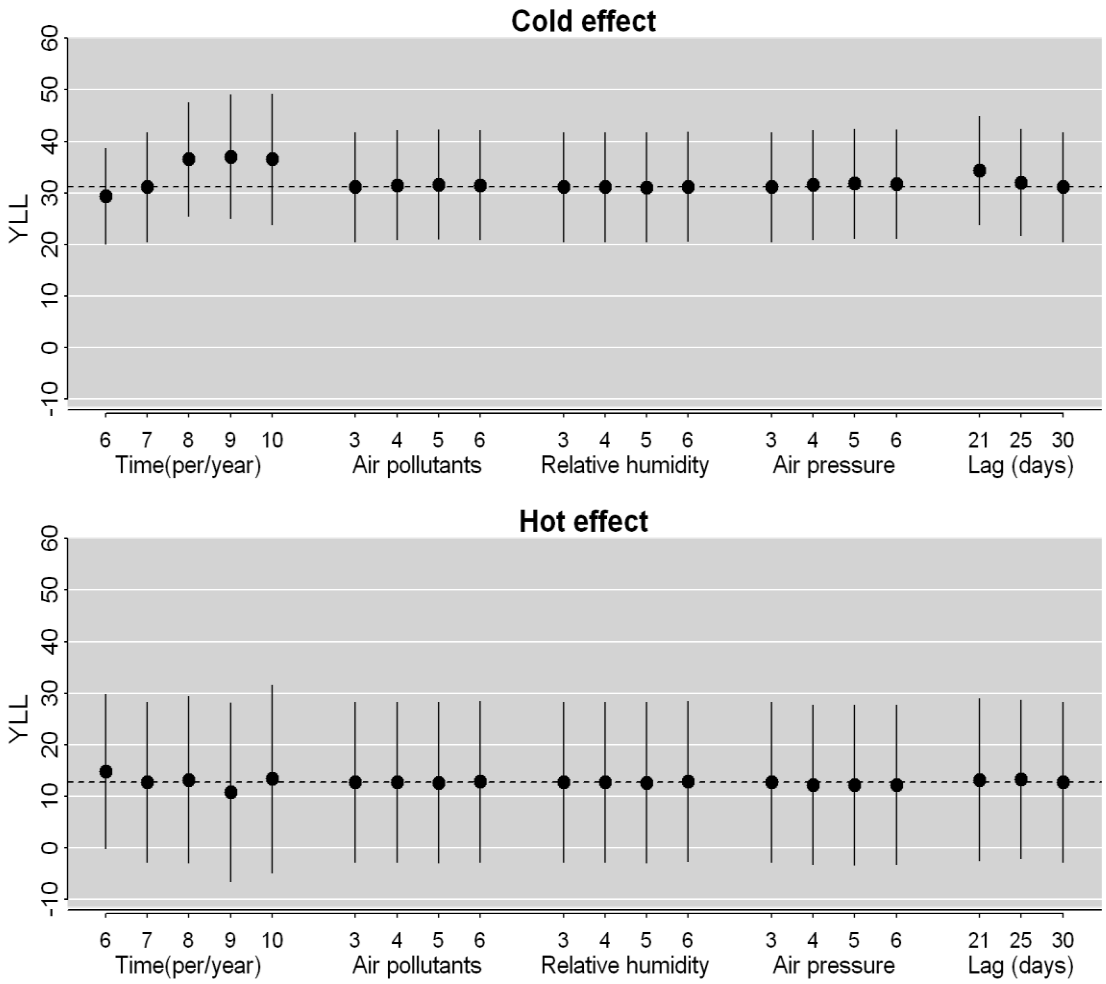

Figure S2: Sensitivity analyses of effect estimates for non-accidental mortality by changing degrees of freedom (df) (6-10 per year) for time to control for season, df (3-6) for air pollutants, relative humidity and atmospheric pressure and maximum lag from 21 to 30 days for mean temperature.

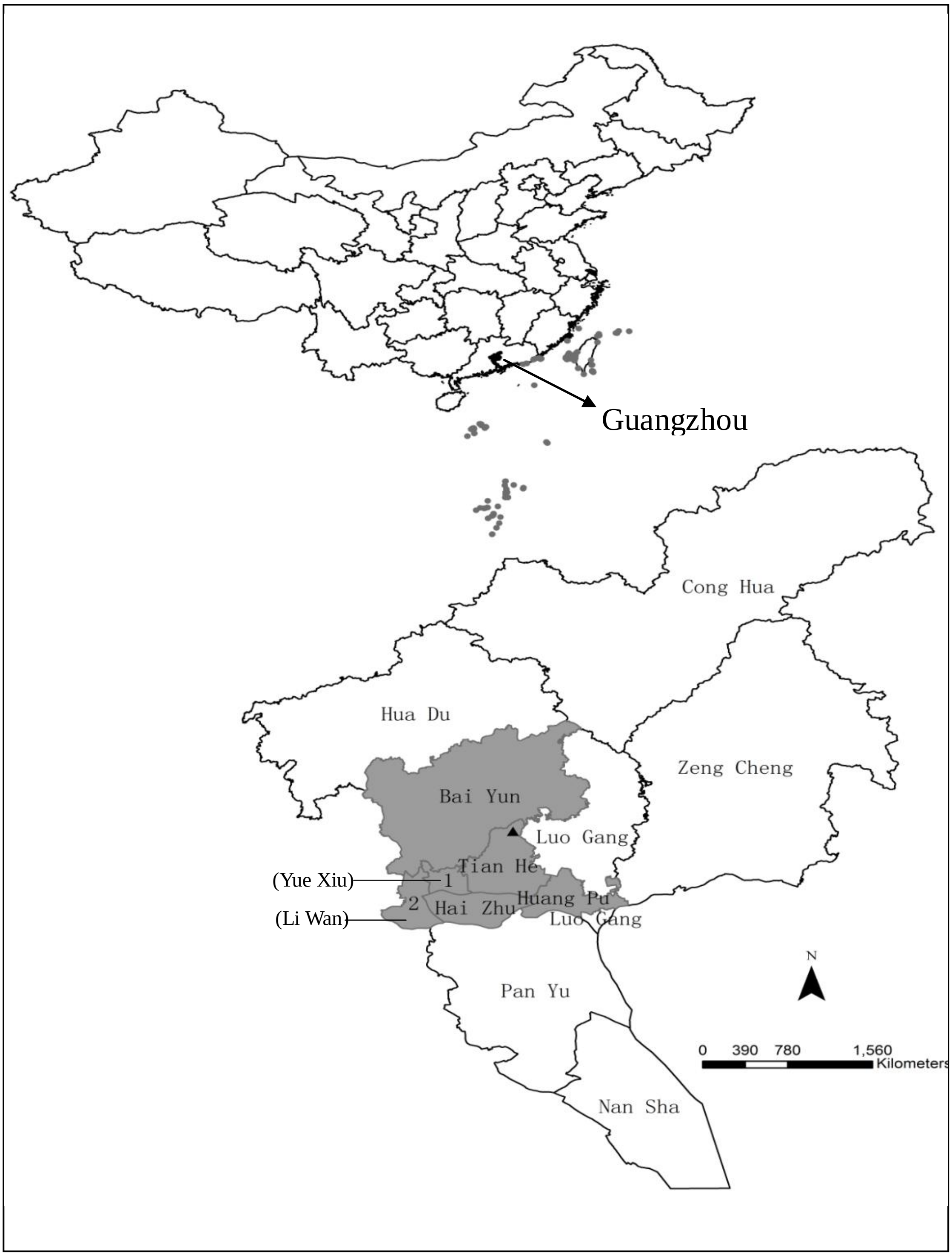

Figure S3: Map of districts in Guangzhou, China. The grey areas are the six central urban districts of Guangzhou under study. The triangle shows the location of Guangzhou Weather Station. This map was generated by ArcGIS software, version 10.1.

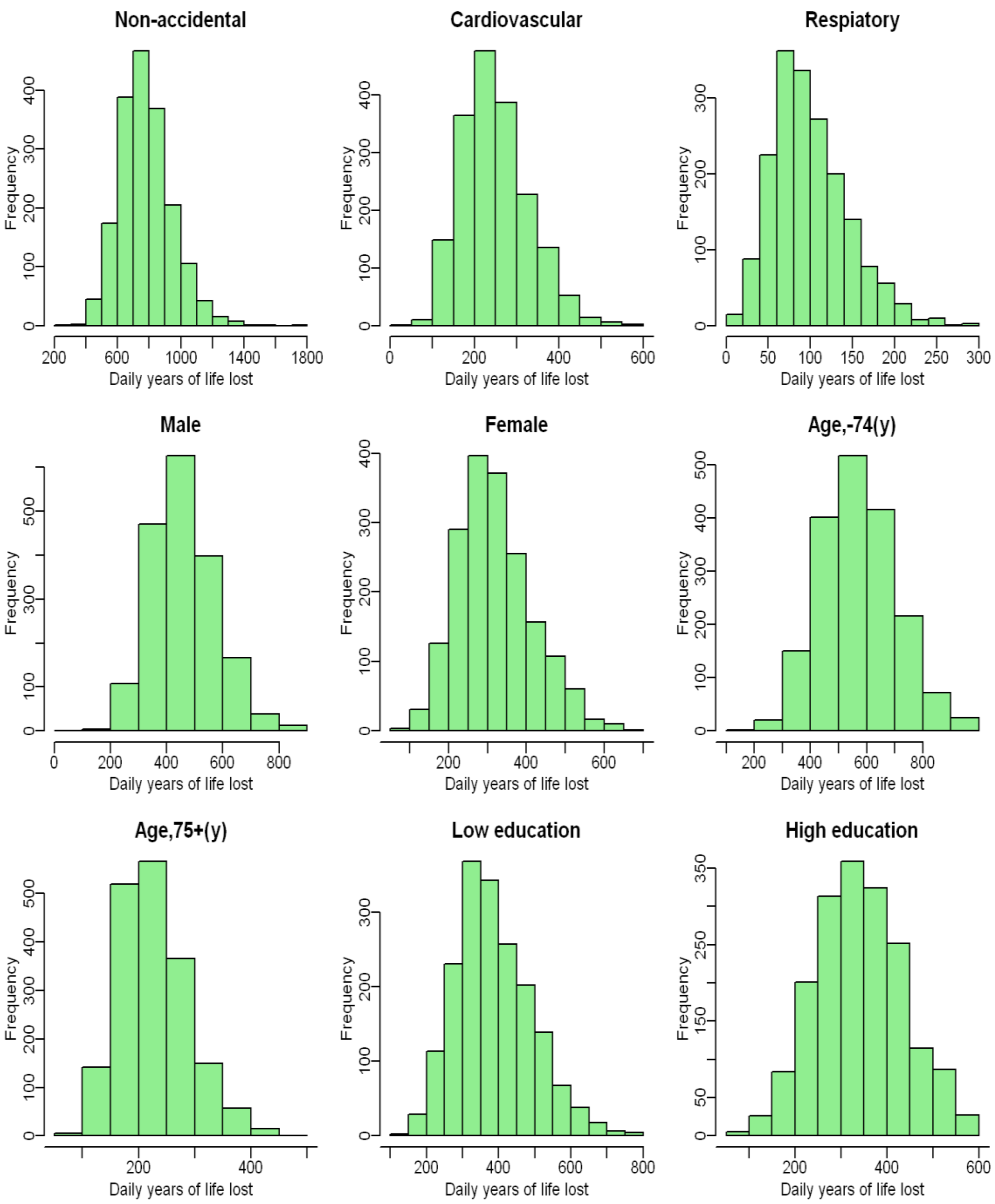

Figure S4: Histogram of daily years of list lost due to mortality categories in Guangzhou, China during 2003-2007.
